# Prevalence of Type 1 Diabetes Among People Aged 19 and Younger in the United States

**DOI:** 10.5888/pcd15.180323

**Published:** 2018-11-21

**Authors:** Mary A.M. Rogers, Benjamin S. Rogers, Tanima Basu

**Affiliations:** 1 Department of Internal Medicine, University of Michigan, Ann Arbor, Michigan; 2 Institute of Healthcare Policy and Innovation, University of Michigan, Ann Arbor, Michigan; 3 Department of Geography, Bowling Green State University, Bowling Green, Ohio

**Figure:**
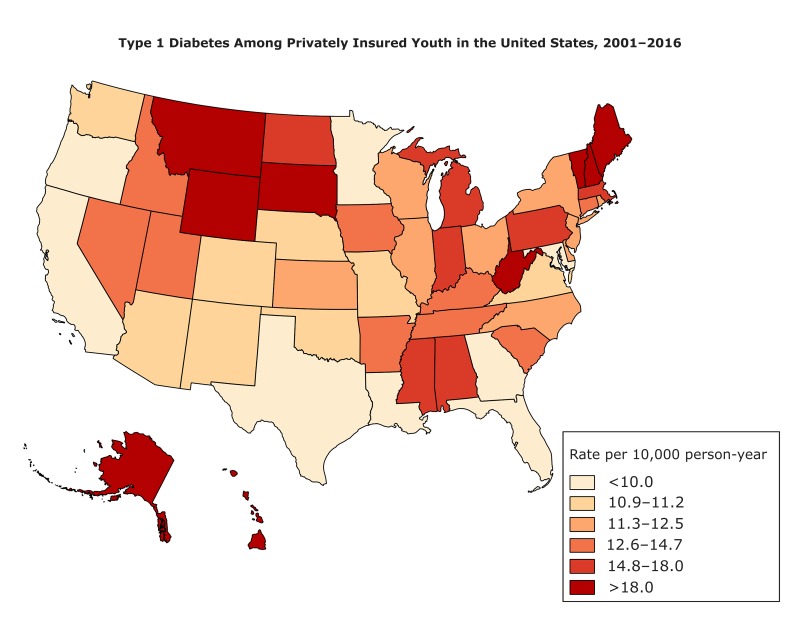
Prevalence rate per 10,000 person-years of type 1 diabetes among people aged 19 or younger with private health insurance, by state, 2001–2016. Rates were mapped by quantiles (frequency distribution with equal groups). Rates were highest in Vermont, Hawaii, Maine, Alaska, and Montana. The lowest rates were in California, the District of Columbia, Maryland, Texas, and Louisiana. Data source: Clinformatics Data Mart Database (OptumInsight), Eden Prairie, Minnesota. **Table:** 

Rate Per Person-Year	States
<10.0	California, District of Columbia, Maryland, Texas, Louisiana, Georgia, Minnesota, Oregon, Florida
10.0–11.2	Virginia, Oklahoma, New Mexico, Arizona, Missouri, Nebraska, Colorado, Washington
11.3–12.5	Wisconsin, New York, North Carolina, Illinois, New Jersey, Ohio, Rhode Island, Delaware, Kansas
12.6–14.7	Arkansas, Utah, Tennessee, Connecticut, Iowa, Nevada, Idaho, Kentucky, South Carolina
14.8–18.0	Massachusetts, Mississippi, Indiana, North Dakota, Michigan, Alabama, Pennsylvania
>18.0	West Virginia, New Hampshire, Wyoming, South Dakota, Montana, Alaska, Maine, Hawaii, Vermont

## Background

Large national surveys that use telephone or in-person interviews have been the source of population-based estimates of diabetes prevalence (1,2). Such surveys in the United States usually do not distinguish between types of diabetes; therefore, maps of type 1 diabetes have been difficult to generate. The advent of large, nationwide databases from health insurers has enabled researchers to investigate geographic variations in disease among the privately insured population. By using such a database, we designed an epidemiologic study to examine the prevalence of type 1 diabetes among people aged 19 or younger across all 50 states and Washington, DC.

## Data Sources and Map Logistics

We used data from January 1, 2001, through June 30, 2016, from the Clinformatics Data Mart Database (OptumInsight). This nationwide database contains integrated longitudinal health information on 73 million Americans with private health insurance, including demographic data, membership information, prescription medications, and outpatient and inpatient services.

We determined eligibility criteria for type 1 diabetes by using a validated procedure (3). First, data on people with a ratio of 0.6 or more type 1 diabetes diagnoses to type 2 diagnoses were extracted from inpatient and outpatient files. This algorithm had a positive predictive value of 98.7% for detecting type 1 diabetes (3). Second, people without any type 2 diabetes diagnosis and with only type 1 diagnoses were extracted; this algorithm had a positive predictive value of 99.3% for ascertaining type 1 diabetes (3).

We had no sex or racial/ethnic restrictions. We included only people aged 19 or younger at the time of enrollment in a health insurance plan. Rates were calculated as the total number of diagnoses of type 1 diabetes in a state from 2001 through 2016 (numerator) divided by the person-years of the underlying insured members in each state during the same period (denominator). Prevalence rates were expressed as cases (both existing and incident) per 10,000 person-years. Because this database constitutes a sample of people with private health insurance in each of the 50 states and the District of Columbia, we estimated the number of people aged 19 or younger with type 1 diabetes in the reference population (privately insured) for each state in 2015 by using the state-specific prevalence rates and the number of people aged 19 or younger with private health insurance in each state (4). Analyses were conducted by using Stata/MP version 15.1 (StataCorp LLC) and mapped by using QGIS Geographic Information System, version 2.18 (QGIS.org).

## Highlights

In our nationwide sample of people covered by private health insurance from 2001 through 2016, we identified 45,047 people aged 19 or younger who had type 1 diabetes. Vermont had the highest prevalence rate of type 1 diabetes (79.6/10,000 person-years) followed by Hawaii, Maine, Alaska, Montana, South Dakota, Wyoming, and New Hampshire (Table). The lowest prevalence rates of type 1 diabetes among people aged 19 or younger were in California, the District of Columbia, Maryland, Texas, and Louisiana. We found a 14.7-fold difference in prevalence rates across all 50 states (79.6/5.4). States with large populations had the greatest number of privately insured young people with type 1 diabetes, with Pennsylvania, Texas, New York, California, Michigan, Illinois, Florida, and Ohio ranking the highest (Table).

**Table:** Prevalence Rate and Number of People Aged 19 or Younger With Type 1 Diabetes, Ranked by State, United States 2001–2016

Prevalence Rate per 10,000 Person-Years, 2001–2016			Number, 2015^a^	
Rank	State	Rate (95% Confidence Interval)	State	Number
1	Vermont	79.6 (43.5–133.6)	Pennsylvania	3,540
2	Hawaii	41.5 (15.2–90.3)	Texas	3,480
3	Maine	40.0 (29.3–53.4)	New York	3,230
4	Alaska	27.5 (18.0–40.3)	California	3,030
5	Montana	26.8 (19.5–35.9)	Michigan	2,450
6	South Dakota	22.5 (16.7–29.8)	Illinois	2,360
7	Wyoming	20.7 (15.5–27.1)	Florida	2,250
8	New Hampshire	18.6 (15.4–22.3)	Ohio	2,230
9	West Virginia	18.2 (14.9–22.1)	New Jersey	1,770
10	Pennsylvania	17.8 (16.5–19.2)	Indiana	1,640
11	Alabama	16.4 (14.8–18.0)	Massachusetts	1,630
12	Michigan	15.7 (14.6–16.8)	North Carolina	1,570
13	North Dakota	15.3 (11.8–19.4)	Georgia	1,390
14	Indiana	14.9 (13.9–15.9)	Virginia	1,370
15	Mississippi	14.9 (13.2–16.6)	Tennessee	1,250
16	Massachusetts	14.8 (13.5–16.1)	Washington	1,220
17	South Carolina	14.7 (13.2–16.3)	Wisconsin	1,130
18	Kentucky	14.7 (13.5–15.9)	Alabama	1,110
19	Idaho	14.6 (12.4–17.1)	Arizona	1,080
20	Nevada	14.6 (12.9–16.4)	Missouri	1,060
21	Iowa	13.8 (12.5–15.3)	Utah	1,010
22	Connecticut	13.6 (12.3–15.0)	Minnesota	990
23	Tennessee	13.3 (12.4–14.2)	South Caroline	970
24	Utah	13.2 (12.3–14.1)	Colorado	960
25	Arkansas	12.7 (11.3–14.2)	Kentucky	930
26	Kansas	12.5 (11.4–13.7)	Hawaii	890
27	Delaware	12.4 (9.3–16.2)	Maryland	810
28	Rhode Island	12.2 (11.1–13.3)	Connecticut	790
29	Ohio	11.9 (11.5–12.3)	Iowa	780
30	New Jersey	11.8 (11.1–12.5)	Maine	730
31	Illinois	11.7 (11.1–12.3)	Kansas	660
32	North Carolina	11.7 (11.1–12.2)	Vermont	640
33	New York	11.3 (10.6–11.9)	Nevada	640
34	Wisconsin	11.3 (10.7–11.8)	Louisiana	590
35	Washington	11.2 (10.2–12.2)	Oklahoma	580
36	Colorado	11.1 (10.6–11.6)	Oregon	550
37	Nebraska	10.7 (9.8–11.7)	Mississippi	520
38	Missouri	10.7 (10.2–11.2)	Arkansas	460
39	Arizona	10.6 (10.0–11.1)	Idaho	430
40	New Mexico	10.5 (9.0–12.2)	West Virginia	420
41	Oklahoma	10.3 (9.5–11.2)	Montana	410
42	Virginia	10.0 (9.4–10.6)	New Hampshire	410
43	Florida	9.8 (9.5–10.1)	Nebraska	390
44	Oregon	9.7 (8.6–10.8)	South Dakota	330
45	Minnesota	9.7 (9.2–10.1)	Alaska	290
46	Georgia	9.5 (9.1–9.9)	New Mexico	250
47	Louisiana	9.4 (8.7–10.2)	North Dakota	220
48	Texas	8.5 (8.3–8.7)	Wyoming	220
49	Maryland	8.4 (7.9–8.9)	Rhode Island	190
50	District of Columbia	6.0 (4.4–8.0)	Delaware	180
51	California	5.4 (5.2–5.6)	District of Columbia	<100

a Estimated number of people aged 19 or younger with type 1 diabetes and private health insurance in 2015.

## Action

Public health efforts to prevent disease and develop interventions often begin with an assessment of where the disease occurs. We conducted a large, nationwide assessment of the prevalence of type 1 diabetes among young people with private health insurance in the United States. We found considerable variation in the prevalence rate of type 1 diabetes across the 50 states, with a nearly 15-fold difference from the highest to lowest prevalence rates. Previously, data from the National Health and Nutrition Examination Survey were used to estimate the prevalence of type 1 diabetes, but with a sample of 123 people with the disorder aged younger than 30, precise state-specific rates could not be calculated (5). In the SEARCH for Diabetes in Youth study, data were collected from locations in only 5 states and from selected Native American sites, not for all 50 states (6). Although our study does include all 50 states, it is important to note that these data represent only children and adolescents with private health insurance. Additional data are needed to assess geographic variation among young people with public health insurance.

Our results suggest that geographic variation in the prevalence rate of type 1 diabetes among young people is different from that of type 2 diabetes (2). Although genetic predisposition plays a role in both types, precipitating factors vary, with autoimmune-related factors being closely associated with type 1 diabetes and lifestyle factors associated with type 2 diabetes (2). The availability of health services, however, is critical for people with either type to prevent long-term complications.

The Patient Protection and Affordable Care Act included provisions to enable people with pre-existing conditions to secure health insurance, which has important implications for those with diabetes (7). The most frequent barriers to health care among young people with type 1 diabetes are cost, communication problems, and obtaining needed information (8). Insurance alone does not eliminate all such barriers but should curtail some, such as cost, although interruptions in insurance remain a concern (9). The frequency of such interruptions varies by state and is associated with 5-fold increases in emergency department visits and hospitalizations (9).

The variation in state-specific prevalence rates of type 1 diabetes is mirrored by state-level variability in services. Not all states mandate that insurers cover diabetes treatment and supplies (10). Alabama, Idaho, North Dakota, and Ohio do not have such mandates. Missouri also does not have a mandate across all insurance policies but requires that insurers offer at least one policy that covers treatment of diabetes (10). Laws relevant to emergency access to insulin also differ; 10 states now allow pharmacists to dispense insulin with an expired prescription in emergency situations. Therefore, one actionable consequence of our study would be to improve state laws and consider federal legislation so that patients with type 1 diabetes are provided the services necessary for optimal health — regardless of the state in which they live.
